# Astrocytes in intracerebral hemorrhage: impact and therapeutic objectives

**DOI:** 10.3389/fnmol.2024.1327472

**Published:** 2024-02-14

**Authors:** Hao Dong, Xin Wen, Bai-Wen Zhang, Zhe Wu, Wei Zou

**Affiliations:** ^1^The First Clinical Medical College, Heilongjiang University of Chinese Medicine, Harbin, China; ^2^The Third Department of Acupuncture and Moxibustion, First Affiliated Hospital, Heilongjiang University of Chinese Medicine, Harbin, China

**Keywords:** astrocytes, crosstalk, inflammatory response, intracerebral hemorrhage, neurological function

## Abstract

Intracerebral hemorrhage (ICH) manifests precipitously and profoundly impairs the neurological function in patients who are affected. The etiology of subsequent injury post-ICH is multifaceted, characterized by the intricate interplay of various factors, rendering therapeutic interventions challenging. Astrocytes, a distinct class of glial cells, interact with neurons and microglia, and are implicated in a series of pathophysiological alterations following ICH. A comprehensive examination of the functions and mechanisms associated with astrocytic proteins may shed light on the role of astrocytes in ICH pathology and proffer innovative therapeutic avenues for ICH management.

## 1 Introduction

Intracerebral hemorrhage (ICH) is a common subtype of fatal stroke. In treatment, mainly supportive medications are used, while surgical removal of hematomas remains controversial (Liao et al., 2017). Astrocytes, a type of abundant and complex shaped glial cells, play a role in the central nervous system. Astrocytes form functional networks in the brain through gap connections and transmit information to each other (Sofroniew and Vinters, 2010). Astrocytes not only modulate the neuronal microenvironment, constitute elements of the blood-brain barrier (BBB), and provide energy to neurons but are also involved in glutamate uptake, immunoregulatory activities, and synaptic modulation (Endo et al., 2022). Astrocytes exhibit a response called astrocyte reactivity to central nervous system (CNS) damage. This reactivity leads to changes in the cellular morphology and molecular expression (Sofroniew, 2020). Reactive astrogliosis is a common response of astrocytes to brain injury and various diseases, such as trauma, infection, neurodegeneration, and ischemia (Zamanian et al., 2012).

The secondary brain injury after ICH is mainly caused by edema, inflammation, and the metabolic effects of clot components (Wilkinson et al., 2018). The peri-hematomal region undergoes a series of secondary brain pathologies encompassing the disruption of the blood-brain barrier, the induction of oxidative stress, inflammatory cascades, programmed cell death, activation of glial cells, and perturbations in cerebral energy metabolism. These concurrent pathophysiological processes can collectively intensify cerebral damage, making treatment difficult. Studying the function of astrocytes may provide new therapeutic ideas for the disruption of the blood-brain barrier (BBB), inflammatory response, and neuronal cell death after ICH.

## 2 Astrocytes in intracerebral hemorrhage

### 2.1 Astrocytes and inflammatory response

Astrocytes have pro-inflammatory and anti-inflammatory functions, depending on the mode of injury. Neuroinflammation and ischemia induce two different types of reactive astrocytes, known as “A1” and “A2,” respectively. A1 may have “harmful” functions, while A2 may have “beneficial” or repair functions (Liddelow and Barres, 2017). Inflammatory response is a key factor in ICH and can progress under various factors (Zhou et al., 2014).

This response is intrinsically tied to the activation of glial cells, the infiltration of peripheral inflammatory cells into cerebral tissue, and a subsequent increase in the levels of pro-inflammatory cytokines and chemokines. Such activities culminate in the onset of cerebral edema, cellular apoptosis, and notable neurological impairments. Researches have shown that toll-like receptor-4/nuclear factor kappa-light-chain-enhancer of activated B cells (TLR-4/NF-κB) pathway and reactive oxygen species/mitogen-activated protein kinase/nuclear factor E2-related factor 2 (ROS/MAPK/Nrf2) pathway can mediate inflammatory response after ICH (Zhang and Zhang, 2018; Wu et al., 2022). Notably, astrocytes possess the capacity to detect and react to pro-inflammatory cells (Xie et al., 2020).

The Janus kinase/signal transducer and activator of transcription 3 (JAK/STAT3) pathway, the nuclear factor kappa-light-chain-enhancer of activated B cells (NF-κB) pathway and the mitogen-activated protein kinase (MAPK) pathway can modulate and control astrocyte reactivity. Multiple pathways crosstalk produce an effect on the phenotype of reactive astrocytes (Giovannoni and Quintana, 2020). In addition, during the inflammatory process, astrocyte signaling pathway may converge on common downstream transcription regulatory factors. The central step in astrocyte activation is nuclear translocation of the NF-κB heterodimer (Linnerbauer et al., 2020). Therefore, exploring the role of astrocytes in the inflammatory response can help become a therapeutic target for neuroinflammation in ICH.

#### 2.1.1 S100B

S100B belongs to the mutagenic family and can affect the activation of the glial fiber acidic protein (GFAP), reactive oxygen species (ROS) release, and mitochondrial dysfunction (Langeh and Singh, 2021). S100B is expressed primarily by astrocytes (Cordeiro et al., 2022).

S100B overexpression has been linked to an increased production of pro-inflammatory cytokines and the facilitation of neuronal apoptosis. In the aftermath of ICH, there is an upsurge in the expression of the S100B protein. This coincides with the activation of astrocytes and resident microglia, resulting in the release of pro-inflammatory cytokines and ROS, further amplifying neuroinflammatory processes. Aromatic acid (AA) serves as an inhibitor to the synthesis of S100B within astrocytes. Subsequent to AA administration, there is a marked decrease in both peripheral and central S100B levels. Additionally, AA mitigates excessive astrogliosis, curtails microglial activation, and reduces cellular apoptosis, ROS release, and the secretion of inflammatory mediators such as interleukin-1β (IL-1β) and tumor necrosis factor-α (TNF-α). Consequently, AA has demonstrated efficacy in counteracting motor dysfunction observed in ICH-afflicted rats (Cordeiro et al., 2020, 2022).

#### 2.1.2 NLRP6

Innate immunity plays a role as a self-defense mechanism in CNS damage. The inflamasome is an intracellular immune sensor that regulates immune and inflammatory responses (Shao et al., 2018). Nucleotide-binding and oligomerization domain (NOD)-like receptor (NLR) family activate inflammasome signaling. The NOD-like receptor family pyrin domain containing 6 (NLRP6) is one of the members of the NLR family, capable of regulating neuroinflammation and promoting the recovery of damaged peripheral nerves (Ghimire et al., 2020).

The expression of the NLRP6 inflammasome protein escalates following 6 h post-ICH, reaching its peak at the 1-day mark. Concomitantly, at this 1-day post-ICH interval, there is a proliferation of NLRP6-positive cells in the vicinity of the hematoma, predominantly localizing within astrocytes. A comparative analysis between wild-type (WT) and NLRP6-deficient (Nlrp6^−/−^) mice reveals heightened activities of IL-1β, IL-6, TNF-α, and NF-κB in the latter. This suggests that the increased expression of astrocyte-sourced NLRP6 inflammasome exerts a neuroprotective influence on cerebral structures. Conversely, the absence of the NLRP6 inflammasome may amplify the severity of brain injury subsequent to ICH (Wang et al., 2017). Intriguingly, Tlr4^−/−^ mice exhibited reduced NLRP6 expression when contrasted with WT mice, a finding that aligns with the results observed with the TLR4 antagonist TAK242 (Wang et al., 2017). NLRP6 inflammasome has a protective effect on brain injury after ICH.

#### 2.1.3 Prdx1

Posttranscriptional regulation plays a pivotal role in various pathophysiological mechanisms. For example, exploring the blood transcriptome after cerebral hemorrhage can help understand immune and coagulation pathways and identify biomarkers (Stamova et al., 2019).

Peroxiredoxin 1 (Prdx1), an integral member of the peroxiredoxin family, exhibits an enhanced expression in proximity to the hematoma subsequent to ICH. This elevated expression is observed to peak at the 72-h mark. Notably, Prdx1 co-localizes with both astrocytes and microglia. Experimental introduction of a Prdx1-overexpressing adeno-associated virus into the right striatum led to notable reductions in mortality and hematoma volume. Concurrently, there was a discernible increase in the count of Nissl-positive cells coupled with an augmented expression of the Bcl2/Bax ratio. Additionally, mRNA levels of inflammatory markers, namely TNF-α and IL-6, underwent a decline. In contrast, a significant proportion of Prdx1-knockdown rats succumbed within a 3-day span post-ICH. *In vitro* studies have revealed that the RNAs bound by Prdx1 are predominantly localized within the coding sequence region, the 3′UTR, and the 5′UTR, influencing RNA stability. Prdx1 demonstrates the capacity to bind directly to mRNA transcripts, manifesting both anti-inflammatory and anti-apoptotic properties. Within astrocytes specifically, Prdx1 is identified to associate with the mRNAs of ANGPTL4, GADD45A, and THBS1 (Yang et al., 2020). Transcripts that have recognized roles in inflammatory processes and cellular apoptosis.

#### 2.1.4 TLR2

The heme released by cells is harmful to brain tissue and activates related signaling pathways, inducing ROS production and inflammatory mediators, leading to oxidative damage and inflammation (Martins and Knapp, 2018). Toll-like receptors (TLRs) serve as foundational elements in the upstream immune and inflammatory cascade within the brain. Toll-like receptor 2 (TLR2) is associated with inflammatory response. Following ICH, there is an observed upregulation in the expression of TLR2. This augmented TLR2 expression is associated with a compromise in the integrity of the blood-brain barrier, a consequence of activating matrix metalloproteinases-9 (MMP-9) within astrocytes. Experimental administration of hemin led to an induction of TLR2 expression, and intriguingly, hemin was found to activate the p44/42 MAPK pathway in a TLR2-dependent fashion, which in turn exacerbates secondary brain damage. Consequent to hemin administration, a pronounced accumulation of neutrophils was noted in the afflicted cerebral tissue. In stark contrast, the infiltration of neutrophils was considerably diminished in TLR2 KO mice (Min et al., 2017). These observations lend credence to the hypothesis that heme acts as a stimulant for TLR2 within astrocytes, thereby triggering an inflammatory cascade. As such, the heme-TLR2 signaling axis may be involved in neutrophil infiltration.

#### 2.1.5 CK2

Subsequent to ICH, there is a surge in the levels of glutamate, leading to the activation of NMDAR. N-methyl-D-aspartate (NMDA) receptors constitute the principal subtype of extra-synaptic glutamate receptors. Within the NMDA receptor family, the NR2B subunit is one component of the NR2 subunits, which include NR2A through NR2D. The PDZ ligands present on the NR2B subunit possess the capability to associate with the scaffolding protein, PSD-95, resulting in the formation of the NR2B-PSD95 complex. This complex plays a significant role in modulating downstream synaptic signaling (Lai et al., 2011; Ge et al., 2020). Casein kinase 2 (CK2) is classified as a phosphorylated serine/threonine kinase. Observations have denoted that 24 h post-ICH, there is a decline in CK2 protein levels while NR2B expression witnesses an increase. Remarkably, the overexpression of CK2 facilitates the phosphorylation of NR2B at the S1480 site, leading to a reduction in NR2B expression as well as in the levels of the NR2B-PSD95 complex. This phenomenon is accompanied by decreased nerve damage and cerebral water content. After cultured neurons and astrocytes *in vitro*. *In vitro* experiments have illuminated that CK2 overexpression fosters both the activation and proliferation of astrocytes and concurrently reduces the levels of pro-inflammatory cytokines, TNF-α and IL-6, within these cells. Furthermore, CK2 overexpression offers a protective role against neuronal apoptosis and oxidative stress (Sun et al., 2022). Thus, it is evident that CK2 wields a regulatory influence over astrocyte activation and proliferation, and is instrumental in mediating neuronal apoptosis, the inflammatory cascade, and oxidative damage.

### 2.2 Astrocytes and blood-brain barrier damage

The blood-brain barrier (BBB) plays an important role in maintaining normal neuronal activity and a stable microenvironment, as well as balancing oxygen and ion concentrations (Kadry et al., 2020). Astrocytes are involved in the formation and stability of the BBB, and have potential therapeutic effects on brain edema and BBB damage after ICH.

#### 2.2.1 AQP4

Aquaporin 4 (AQP4) is a member of the aquaporins family, widely expressed in the nervous system (Pan et al., 2022). It is predominantly located in the foot processes of astrocytes surrounding the capillaries and plays a pivotal role in maintaining the integrity of the blood-brain barrier (BBB). This barrier, along with astrocytes, supplies neurons with energy substrates such as glutamate and lactic acid (Bordone et al., 2019).

In pathological states, astrocytes can assume a barrier function, regulating water uptake through a mechanism associated with AQP4 (Haj-Yasein et al., 2011). Post-stroke, AQP4 channels, located in the blood-brain barrier astrocytes facilitate water entry into the central nervous system (CNS), culminating in cerebral edema.

The disruption of the BBB or aquaporins due to ICH gives rise to perihematomal edema (PHE). Harmful substances in the blood can exacerbate delayed PHE through the compromised blood-brain barrier, resulting in increased production of ROS and MMP-9 (Turner and Sharp, 2016). In the early stages of PHE formation, cytotoxic edema is dominant, often evident as astrocyte swelling. PHE accumulation is rapid within 1 to 3 days post-onset and continues to rise gradually up to 12 to 14 days (Wan et al., 2023).

ICH mice exhibit pronounced astrocyte activation, and within the PHE region, AQP4 expression diminishes. Enhancing AQP4 expression can fortify the blood-brain barrier, suggesting it as a potential regulatory target for PHE mitigation post-ICH (Jeon et al., 2021). Tang et al. (2010) illustrated that the absence of AQP4 exacerbated cerebral edema, neurological deficits, and neuronal death.

Vascular endothelial growth factor (VEGF) is integral for angiogenesis and neuroprotection. Upon blood-brain barrier damage, there is a co-localization of VEGF and AQP4 on astrocyte processes (Kaur et al., 2006). Research by Chu et al. highlighted that increased AQP4 expression shields astrocytes and tight junctions post-ICH (Chu et al., 2013). VEGF exerts protective effects on cerebral edema, neurological deficits, and neuronal death around the hematoma after ICH, linked with the upregulation of AQP4 expression and activation of JNK and ERK pathways. Furthermore, a significant count of TUNEL stain-positive cells, primarily neurons and astrocytes, were observed around the hematoma in Aqp4^−/−^ mice. Compared to Aqp4^+/+^ mice, there was a notable elevation in caspase-3 and caspase-8 in AQP4-deficient mice, whereas caspase-9, Bax, and Bcl-2 levels remained unchanged (Chu et al., 2014).

The “Glymphatic” system, a network of perivascular tunnels encased in astrocyte end-feet, is vital for waste removal, substance exchange, and involvement in CNS diseases (Plog and Nedergaard, 2018). Liu et al. (2021a) demonstrated that cervical lymphatic blocking (CLB) post-ICH exacerbated neuronal apoptosis and cerebral edema around the hematoma. This condition upregulated TNF-α while downregulating AQP4, promoting astrocyte activation and inflammation via an imbalance between TNF-α and IL-10. However, the role of AQP4 post-ICH remains contentious. Some researchers have proposed that AQP4 may exacerbate ICH conditions. For instance, Wang et al. observed a significant surge in AQP4 and Aquaporin9 (AQP9) expressions around the hematoma post-ICH (Wang et al., 2015). Post-curcumin administration, cerebral edema and neurological dysfunctions were alleviated. *In vitro* studies demonstrated that Fe^2+^ upregulated NF-κB p65, AQP4, and AQP9 proteins in astrocytes. In contrast, curcumin suppressed AQP4 and AQP9 expressions via the NF-κB pathway, highlighting the potential of curcumin's protective effects post-ICH.

AQP4 mRNA and protein increased after ICH. Carvacrol, a natural compound derived from the Lamiaceae family, was shown by Zhong et al. (2013) to dose-dependently reduce AQP4 mRNA levels, improving neurological deficits, cerebral edema, and downregulating AQP4 protein expression in the perihematomal region. These results suggest that the alleviating effect of carvacrol on cerebral edema may be related to the regulation of AQP4 expression. But Carvacrol may also alleviate brain damage, thereby affecting the expression of AQP4 (Zhong et al., 2013). Further research is needed on how Carvacrol affects the expression of AQP4 in ICH.

#### 2.2.2 EMMPRIN

The CNS is structurally supported by a framework of extracellular matrix (ECM) proteins. One group of enzymes, matrix metalloproteinases (MMPs), plays a pivotal role in the pathological restitution process that ensues following brain trauma. A key regulator that acts upstream of MMPs is EMMPRIN (Extracellular matrix metalloproteinase inducer, also known as CD147). EMMPRIN is instrumental in modulating the expression of MMPs and in maintaining CNS homeostasis. Post-ICH, EMMPRIN expression has been observed to escalate, reaching a peak approximately 3 days after the onset of the hemorrhage. Moreover, its localization has been identified to coincide with both astrocytes and microglia. Experimental interventions involving the injection of anti-EMMPRIN monoclonal antibodies have yielded compelling results: there is a marked reduction in MMP-9 expression and a subsequent mitigation in neutrophil infiltration. These findings suggest that, in the aftermath of ICH, EMMPRIN acts to augment the expression of MMP-9, thereby exacerbating cerebral injury (Liu et al., 2022).

### 2.3 Astrocytes and pathological products after intracerebral hemorrhage

#### 2.3.1 Hepcidin

Iron is a significant hematoma breakdown product after ICH, which can cause DNA damage and cognitive impairment. Hepcidin is a liver-secreted peptide hormone that regulates intracellular iron efflux by binding and internalizing ferroportin, hence influencing plasma iron concentration and systemic iron content (Nemeth et al., 2004; Nemeth and Ganz, 2023).

Toll like receptor 4 (TLR4) expression is upregulated in reactive microglias after ICH, and NF-κB is activated through the myeloid differentiation factor 88/TIR-domain-containing adapter-inducing interferon-β (MyD88/TRIF) signaling pathway (Lin et al., 2012).

Interleukin-6 (IL-6) is a TLR4 signaling-dependent inflammatory cytokine that stimulates STAT3 to control hepcidin (Wrighting and Andrews, 2006). Xiong et al. (2016) demonstrated that the cerebral iron concentration of ICH mice gradually increased, as did the hepcidin, which reached its peak at 3 days. It is predominantly colocalized with astrocytes and minimally expressed by microglia, suggesting that hepcidin is important in iron metabolism in the brain. In Hepc^−/−^ mice, the accumulation of iron and water is reduced, and they display enhanced learning and memory capacities. Human hepcidin-25 peptide causes iron buildup in the brain, oxidative damage, and worsen the cognitive impairment. The TLR4/MyD88 signaling pathway stimulates the phosphorylation of STAT3 and the production of hepcidin. In Tlr4^−/−^ and Myd88^−/−^ mice, the levels of hepcidin, IL-6, and phosphorylated STAT3 are diminished. The serum iron level was lower in Tlr4^−/−^ mice and Myd88^−/−^ mice compared to C57BL/6 mice. The upregulation of hepcidin expression by TLR4/MyD88 signaling may worsen iron buildup, oxidative damage, and cognitive impairment. Using the TLR4 antagonist TAK242 can minimize iron buildup in the brain post-ICH.

#### 2.3.2 GHK

Glycine-histidine-lysine (GHK) is a tripeptide inherent to human physiology and is characterized by its anti-inflammatory, antioxidant, and wound-healing properties (Pickart et al., 2017).

Following ICH, the administration of GHK has been observed to precipitate reductions in neurological deficits, caspase-3 expression, and subsequent apoptosis in rats. Moreover, GHK demonstrates a protective role against hemin-induced apoptosis in SH-SY5Y cells by attenuating the expression of miR-339-5p. The functional target gene of miR-339-5p has been identified as VEGFA mRNA, with miR-339-5p exerting a negative regulatory impact. The interplay between miR-339-5p and VEGFA is pivotal in modulating cellular apoptosis. Further insights from studies postulate a connection between GHK and the p38 MAPK pathway (Park et al., 2016; Lyu et al., 2018). Specifically, GHK appears to inhibit the phosphorylation of p38. This phenomenon suggests that the neuroprotective effects rendered by GHK are mediated through a dual mechanism involving decreased miR-339-5p levels and the p38 pathway (Zhang et al., 2018). Another intriguing dimension of the functionality of GHK is evident post-ICH, where its administration is linked to suppressed expression of MMP-2 and MMP-9, diminished levels of pro-inflammatory cytokines, namely TNF-α and IL-1β, and enhanced TIMP1 expression. This pattern insinuates that the anti-inflammatory properties of GHK post-ICH may be derived from its capacity to recalibrate the metalloprotease/anti-metalloprotease equilibrium. Furthermore, GHK appears to favorably influence astrocyte viability without exerting any discernible impact on their proliferation. An intriguing functional target of miR-146a-3p has been identified as AQP4. An elevation in miR-146a-3p expression invariably leads to a decrement in AQP4 expression. GHK administration augments miR-146a-3p levels while inversely affecting AQP4 expression. Furthermore, existing literature highlights the role of the PI3K/Akt pathway in regulating both Aquaporin 1 (AQP1) and AQP4 (Song et al., 2018). *In vitro* studies have corroborated that GHK modulates miR-146a-3p and concurrently inhibits AQP4 expression in astrocytes, primarily mediated through the PI3K/Akt signaling pathway (Zhang et al., 2020).

#### 2.3.3 NDRG2

Glutamate is a crucial neurotransmitter that facilitates physiological excitatory synaptic transmission. However, its over accumulation can result in excitotoxicity. One mechanism to mitigate this potential excitotoxicity is the efficient clearance of glutamate from the synaptic cleft. Excitatory amino acid transporters (EAATs), which are Na^+^-dependent glutamate transporters, play a pivotal role in the absorption of glutamate from this extracellular space (Magi et al., 2019). The N-myc downstream-regulated gene 2 (NDRG2) is an astrocyte-expressed gene that plays a vital role in both the regulation of astrocyte functionality and the enhancement of glutamate uptake within these cells. During cerebral ischemic events, an excess of glutamate is released from the presynaptic membrane. Concurrently, there is an upregulation of NDRG2 expression in astrocytes, which facilitates interaction with Na^+^/K^+^-ATPase β1. This interaction permits both glutamate and sodium to enter astrocytes via EAAT, ensuring the proper activation of glutamate receptors to sustain excitatory synaptic transmission. Notably, mice lacking NDRG2 (Ndrg2^−/−^) exhibit elevated cerebral interstitial glutamate levels, decreased astrocytic glutamate uptake, and exacerbated cerebral ischemic damage. This underscores the protective role of NDRG2 against glutamate-induced excitotoxicity (Yin et al., 2020). Furthermore, NDRG2 has been shown to repress the function of receptor interacting protein kinase 1 (RIPK1), consequently ameliorating astrocyte necrosis post-cerebral ischemia (Zhu et al., 2020).

Reducing cell death and promoting cell survival contribute to the recovery of neurological function after ICH. Post-ICH, there is a surge in NDRG2 expression, peaking at the 24-h mark, which subsequently recedes. Intriguingly, this expression co-localizes with astrocytes (Gao et al., 2018). In wild-type mice (Ndrg2^+/+^), however, there is a discernible decrease in the expression of astrocytic glutamate transporter 1 (GLT1). *In vitro* analyses reveal that NDRG2 has an affinity for NF-κB, obstructing its nuclear translocation and consequently hampering glutamate uptake (Zhou et al., 2020). By mitigating NDRG2 through the NF-κB/GLT1 signaling cascade, it is possible to curtail the neurotoxic effects of glutamate propagated by astrocytes, thus potentially reducing subsequent brain damage post-ICH.

These opposite experimental results may be related to the pathological changes and experimental models caused by different diseases. This may also be related to the proteins and signal pathways regulated by NDRG2. More studies are still needed to elucidate the role of NDRG2 in stroke.

## 3 Cross talk of astrocytes and other cells in intracerebral hemorrhage

### 3.1 Cross talk of astrocytes and microglia

After ICH, microglia are activated and can transform into two phenotypes under the influence of relevant factors: M1 (pro-inflammatory) and M2 (reparative). The activation of microglia to the M1 phenotype mainly occurs in the acute phase after ICH, exacerbating neuroinflammation and brain edema by releasing pro-inflammatory mediators. After transforming into an anti-inflammatory phenotype, microglia can inhibit inflammation and promote neural recovery (Liu et al., 2021b).

This indicates that regulating the activation and phenotype transition of microglia is of great significance for the treatment of secondary brain injury. In neuroinflammation, microglia can control the function of astrocytes, regulate their response, and release glutamate. On the other hand, molecules released by astrocytes can also control the response of microglia and the expression of related genes (Linnerbauer et al., 2020). In summary, the crosstalk between microglia and astrocytes can regulate cytokine and phenotype transitions, affecting the inflammatory response after cerebral hemorrhage.

#### 3.1.1 IL-15

Interleukin-15 (IL-15) is a pro-inflammatory cytokine that plays a pivotal role in the immune system. Within the CNS injury, IL-15 often demonstrates colocalization with astrocytes. In a study by Shi et al. (2020), post-mortem brain tissues of patients with ICH, as well as 1d ICH-induced mouse models, revealed an upregulated expression of IL-15 within astrocytes. This increase was concomitant with hypertrophy of astrocytes, evident by their enlarged morphology and augmented protrusions. Under physiological conditions, an overproduction of IL-15 does not appear to influence neuroinflammation. However, following ICH induction, GFAP-IL-15^tg^ mice displayed pronounced neurological deficits and exacerbated cerebral edema. At the same time, there was a noticeable rise in microglial populations and heightened expression of their activation markers, including CD86, IL-1β, and TNF-α. Such observations suggest that IL-15 potentially modulates microglia toward a pro-inflammatory phenotype. To encapsulate, IL-15 plays a crucial role in modulating the interplay between astrocytes and microglia. This crosstalk is instrumental in potentiating neural injury post-ICH, primarily by amplifying the inflammatory response.

#### 3.1.2 AQP2

Aquaporin-2 (AQP2) is a member of the Aquaporin (AQP) family. In patients suffering from ICH, serum AQP2 expression levels were found to be diminished when compared to healthy counterparts. Furthermore, a negative correlation was established between AQP2 expression and the Modified Rankin Scale scores, which are used to measure the degree of disability or dependence in daily activities. Within the 12 h, 24 h, and 48 h ICH rat model, there was an observable elevation in AQP2 expression in areas proximal to the hematoma, and it co-localized with both astrocytes and microglia. *In vitro* studies have shed light on the pro-inflammatory properties of AQP2. Specifically, the amplification of AQP2 expression within astrocytes was associated with a corresponding increase in the expression of TLR4 and NFκB-P65. This suggests that AQP2 overexpression acts as a catalyst for the activation of astrocytes, principally via the TLR4/NFκB-P65 signaling pathway (Deng et al., 2022). Moreover, previous research has indicated that inhibiting the TLR4/NFκB-P65 signaling pathway plays a pivotal role in inhibiting apoptosis (Dong et al., 2021).

However, the potentiality of AQP2 regulation as a mechanism to inhibit apoptosis, mediated through this specific signaling pathway, remains a topic yet to be thoroughly investigated.

#### 3.1.3 TIM-3

The T-cell immunoglobulin- and mucin-domain-containing molecule (Tims) family is instrumental in the regulation of CD4^+^ T helper 1 (Th1) cells. Notably, Tim-3 is uniquely localized on Th1 cells, where it acts as an inhibitory regulator of the Th1 response.

Twelve hours post ICH, there is a pronounced surge in TIM-3 expression, with peak levels observable at 24 h post ICH. This expression primarily colocalizes with microglia, while its presence on neurons and astrocytes remains negligible. Notably, the use of Tim-3 targeted siRNA therapies has been shown to enhance neurological function, reduce pro-inflammatory cytokines (IL-1β and IL-17), and mitigate both neuronal degeneration and cerebral edema (Chen et al., 2019). Both microglia and astrocytes exhibit the ability to respond to specific ligands by upregulating the expression of TLRs. In this context, galectins, which are β-Galactoside-binding proteins, serve as key modulators of both innate and adaptive immune responses (Vasta, 2009). Specifically, galectin-9, released by astrocytes, can prompt microglia to produce pro-inflammatory cytokines such as TNF and IL-6, intriguingly, in a manner that is independent of Tim-3. This underscores the role of galectin-9 as a communication signal between astrocytes and microglia, which amplifies inflammatory cytokine production and further propels the neuroinflammatory cascade (Steelman and Li, 2014). Furthermore, the lectin galectin-9 is known to activate Tim-3, and this interaction is linked to the potentiation of TLR signaling pathways. A perturbation in this interaction, specifically when TLR-4 dissociates from Gal-9, triggers the TLR-4 signaling cascade. This, in turn, drives microglia toward adopting a pro-inflammatory M1 phenotype, intensifying the overall neuroinflammatory environment. Therefore, the multifaceted role of Tim-3 in guiding microglial polarization and shaping the inflammatory landscape is evident. Mechanistically, this is thought to be orchestrated through the intricate interplay between Tim-3/Gal-9 and the TLR-4 signaling axes (Chen et al., 2019).

#### 3.1.4 TRPA1

The transient receptor potential ankyrin 1 (TRPA1) is a non-specific transmembrane cation channel that exhibits significant permeability to calcium (Ca^2+^). Post ICH, astrocytes express TRPA1, with concurrent enhanced expression of the glial fibrillary acidic protein (GFAP) in surrounding cells and hematomas, signifying astrocytic proliferation. Laboratory studies have demonstrated that suppressing TRPA1, either through antagonism or genetic knockout, mitigates the astrocytic intracellular calcium surge. In Trpa1^−/−^ mice, there is a notable increase in the fluorescence intensity of S100A10, an A2 astrocyte marker, in proximity to the hematoma. Conversely, inflammation markers such as IL-1β, IL-6, p-ERK, as well as the ratios p-ERK/ERK and p-IKKβ/IKKβ, show decreased levels. Additionally, there is an amplified fluorescence intensity of the M2 microglial marker ARG-1, suggesting an anti-inflammatory phenotype. Behaviorally, Trpa1^−/−^ mice demonstrate superior performance, indicating a protective role for TRPA1 suppression (Xia et al., 2023). In essence, TRPA1 in astrocytes modulates inflammation and neurological deficits, notably by downregulating the MAPK/NF-κB signaling pathway, steering astrocytic phenotypic transition toward the A2 type, and fostering the proliferation of phagocytic microglia.

Furthermore, TRPA1 is implicated in white matter pathologies. Myelin Basic Protein (MBP), Demyelinated Myelin Basic Protein (DMBP), and Neurofilament 200 (NF200) are biomarkers reflective of white matter integrity. In ICH-afflicted mice, a concordant decrease in MBP and NF200, juxtaposed against an elevated DMBP, suggests myelin degradation and compromised neural conductivity. Transmission electron microscopy (TEM) reveals axonal swelling and myelin sheath thinning. Therapeutic intervention using TRPA1 antagonists (HC-030031 and A-967079) ameliorates motor dysfunction, augments forelimb muscular strength, enhances MBP and NF200 levels, and attenuates DMBP fluorescence intensity. TEM analysis also shows reduced demyelination, with HC-030031 administration additionally alleviating axonal edema (Xia et al., 2019). The action mechanism of TRPA1 is postulated to involve Ca^2+^-mediated activation of NADPH oxidase 1 (NOX1), which exacerbates oxidative stress by increasing ROS production. Ca^2+^ also activates Calpain1, implicated in myelin degradation (Liu et al., 2006; Baraban et al., 2018). After ICH, both NOX1 and Calpain1 expression surge, but their levels decline with TRPA1 antagonist treatment. This suggests the potential of TRPA1 blockers in counteracting oxidative stress and mitigating white matter injury, specifically myelin damage (Xia et al., 2019).

### 3.2 Cross talk of astrocytes and neurons

In ICH, the communication between astrocytes and neurons is mainly manifested in mitochondrial exchange, uptake and release of glutamate, and glial gap junctions.

Mitochondria, as the power source of cells, play an important role in cellular energy homeostasis and cell fate (Liu et al., 2018). In the brain, cells can exchange mitochondria and conduct signal transduction to enhance cellular function and degrade dysfunctional mitochondria. There is bidirectional mitochondrial transfer between neurons and astrocytes. Neurons can transfer damaged mitochondria to adjacent astrocytes, while astrocytes can release functional mitochondria (Mt) to damaged neurons to enhance antioxidant defense and neuroplasticity (Fairley et al., 2022; Tashiro et al., 2022). The mitochondrial transfer between glial cells and neuronal cells is mainly achieved through the release of extra cellar vessels, formation of tunneling nanotubes, and gap junctions (Fairley et al., 2022). Mitochondrial damage is involved in secondary brain injury after cerebral hemorrhage. Oxidative stress, Mitochondrial DNA (mtDNA) damage, and Calcium (Ca^2+^) overload lead to mitochondrial dysfunction. Mitochondrial dysfunction can cause neuronal apoptosis, white matter damage, demyelinating changes, axonal damage, and immune inflammation (Li et al., 2022). Mitochondrial transfer of astrocytes helps to maintain mitochondrial function and alleviate secondary brain injury after ICH.

Glutamate released from synapses has the potential to interact with group I metabotropic glutamate receptors (mGluRs), subsequently modulating intracellular Ca^2+^ dynamics. An excess or local release of glutamate prompts astrocytes to respond to this synaptic activity (Honsek et al., 2012). Neurons and astrocytes engage in a bidirectional communication mediated by glutamate. Astrocytes can uptake most of the glutamate released from synapses through glutamate uptake transporters to prevent glutamate excitotoxicity. Extra cellar glutamate can promote the release of Calcium (Ca^2+^) from astrocytes, thereby allowing astrocytes to release glutamate to adjacent neurons through exokinetic mechanisms (Mahmoud et al., 2019). In ICH, dysregulation of glutamate-gated channels and sulfonylurea 1 transient receptor potential melastatin 4 is involved in blood-brain barrier disruption and neurotoxicity (Zhang et al., 2023).

Therefore, exploring the role of astrocytes in maintaining glutamate homeostasis may help alleviate neurotoxicity, oxidative stress, and neuronal survival in ICH. Glial cells exhibit high levels of expression of gap junction protein subunits, termed connexins (CXs). The channels formed by these proteins play a significant role in facilitating interactions between glial cells and modulating neural activity (Giaume et al., 2013). The Cx gap junction in glial cells establishes a connection between astrocytes and oligodendrocytes, forming a unified glial network. This network aids in the removal of excitatory, toxic ions, and metabolites. Furthermore, these glial gap junctions advance the propagation of Ca^2+^ slow-waves in astrocytes, which in turn suppresses neuronal hyperexcitability (Lapato and Tiwari-Woodruff, 2018). Regulating the gap junction protein of astrocytes to exert neuroprotective effects can improve brain injury after ICH.

#### 3.2.1 Astrocytic mitochondria

Manganese superoxide dismutase (Mn SOD) serves as a vital antioxidant enzyme, with a primary role in the dismutation of ROS. A deficiency in Mn-SOD can result in excessive ROS accumulation, which has been associated with neuronal death and the manifestation of various neuronal aberrations (Jung et al., 2009). Research conducted by Tashiro et al. (2022) underscored the correlation between enhanced oxidative damage, following ICH, and a decline in the levels of the mitochondrial enzyme Mn SOD. By facilitating the transfer of Mt originating from astrocytes, the Mn-SOD concentrations in male mice were rejuvenated, with a consequent mitigation of neurological impairments. *In vitro* analyses bolstered this observation by revealing that Mt from astrocytes can counteract oxidative stress and avert neuronal death. Mechanistically, this neuroprotection is ascribed to the promotion of neuronal outgrowth, an upregulation of genes associated with synaptogenesis, and a restoration of neuronal Mn-SOD concentrations. Thus, this adaptive transfer of astrocytic Mt augments the neuroprotective antioxidant defenses mediated by neuronal Mn-SOD, simultaneously enhancing neuroplasticity.

#### 3.2.2 HN

Astrocytes possess the capability to acquire and incorporate damaged Mt from neurons. This dynamic interchange amplifies post-ICH, where astrocytes export extracellular mitochondrial particles to neurons. This exchange is orchestrated via CD38-mediated mechanisms, particularly involving CD38/cyclic ADP ribose signaling. This cellular dialogue not only modulates survival pathways but also promotes neuroprotection (Hayakawa et al., 2016). It is noteworthy that, following ICH, there is a marked decline in the expression levels of antioxidant enzymes, Mn SOD and CuZn SOD (Wu et al., 2002).

The mitochondrial DNA (mtDNA)-encoded polypeptide, humanin (HN), boasts cytoprotective attributes. It aids in replenishing mitochondrial glutathione (GSH) levels and counters apoptosis that is precipitated by endoplasmic reticulum stress (Matsunaga et al., 2016). Mt disseminated by astrocytes serve as transport vehicles for HN, ferrying it in various forms including DNA, mRNA, and proteins. Post-ICH events lead to a substantial drop in HN mRNA concentrations as well as its immunoreactivity at the lesion site. However, exogenous HN administration has been shown to be beneficial, as it ameliorates neurological deficits in ICH-afflicted mice and hastens the resolution of hematomas. Astrocytes, as evinced by *in vitro* studies, emerge as a pivotal reservoir of HN. Astrocytic Mt, once secreted, can be integrated by microglial cells, maintaining their structural and functional integrity for up to 72 h. This mitochondrial transfer culminates in elevated intracellular HN concentrations. In the aftermath of ICH, the transcription factor PPARγ plays a cardinal role in guiding the phenotypic transformation of microglia. Concurrently, Mn-SOD acts as a linchpin in sustaining the restorative microglial phenotype. Either HN or mitochondria can amplify the PPARγ/Mn-SOD expression, attenuate pro-inflammatory cytokine levels (such as IL-1β), promote anti-inflammatory responses, and potentiate microglial phagocytosis of red blood cells. It is worth noting that neither HN nor Mt significantly influence microglial proliferation rates. Intriguingly, Mt emanating from HN-deprived astrocytes fail to instigate the reparative microglial phenotype. This underscores the hypothesis that mitochondrial-transported HN is quintessential in promoting the phagocytic or reparative microglial phenotype, which in turn is crucial in rejuvenating neurological function post-ICH (Jung et al., 2020).

#### 3.2.3 Homer1

The Homer/Vesl protein family plays a multifaceted role by modulating the trafficking of type I metabotropic glutamate receptors (mGluRs), influencing axonal guidance, and coordinating the coupling of mGluR to calcium and potassium channels (Ehrengruber et al., 2004). Particularly noteworthy is the interaction between the metabotropic glutamate receptor 5 (mGlu5) and Homer, given its ramifications for Ca^2+^ homeostasis (Ango et al., 2001). Within astrocytes, the dynamics of intracellular calcium signaling, a foundational aspect of gliotransmission, is influenced by mGlu5 activation and its interaction with the Homer1 scaffold protein. Research by Buscemi et al. (2017) unveiled that Homer1a, a short dominant-negative splice variant, sees upregulation in reactive astrocytes. Notably, Homer1a disrupts the mGlu5 and endoplasmic reticulum (ER) interaction, attenuates the magnitude of Ca^2+^ signaling, and consequently curtails glutamate release by astrocytes. Counteracting this upregulation reinstates mGlu5-mediated Ca^2+^ signaling and glutamate release.

Another study shed light on the temporal dynamics of Homer1 protein expression post-ICH in mice (Fei et al., 2022). A conspicuous escalation in Homer1 levels was observed, peaking on the third day post-ICH, and was predominantly co-localized with astrocytes. Around this same temporal landmark, a phenotypic shift of astrocytes from the A1 to the A2 state was discerned. The expression of C3 (a marker of A1 type astrocytes) protein increased on day 1 and 3, while the expression of S100A10 (a marker of A2 type astrocytes) protein increased on day 3 and 7. Overexpressing Homer1 nudged the pro-inflammatory A1 astrocytes toward the anti-inflammatory A2 phenotype, which, in turn, tempered the inflammatory response post-ICH. These astrocytic dynamics and regulations appear to be heavily influenced by the MAPK pathway, as evidenced by heightened ratios of P-Raf-1^*Ser*338^/Raf-1, P-MEK1/2/MEK1/2, and P-ERK1/2/ERK1/2, implying that Homer1 overexpression can ameliorate ICH outcomes by tempering MAPK pathway activation. Direct administration of Homer1 protein resulted in diminished levels of pro-inflammatory markers IL-1β, TNF-α, and C3, while elevating the expression of S100A10. Cerebrospinal fluid analyses further revealed an upregulation in TWEAK, activin A, and persephin, but a downregulation in TNFSF10. Clinically, these findings suggest that harnessing the therapeutic potential of the Homer1 protein could dampen inflammatory responses, conferring neuroprotection. The underlying mechanisms likely involve interplay with molecular entities like TWEAK, activin A, TNFSF10, and persephin.

#### 3.2.4 Connexin 43 (Cx43)

Connexin 43 (Cx43) is a predominant gap junction protein in astrocytes. Cx43 witnesses an upsurge in its expression post-ICH, starting from 6 h and peaking around 72 h. Astrocytes interface with their external environment via gap junction channels (GJCs), which consist of two adjacent half-channels (termed hemichannels, Hcs) situated between adjacent cells. Events like strokes can trigger excessive opening of these Hcs, leading to the release of detrimental substances. Following ICH, there is an elevation in Hcs activity, also peaking at approximately 72 h. Gap19, a specific blocker for Cx43Hcs, when administered, diminishes apoptotic neuronal counts, reduces hematoma volume, and curtails the inflammatory response. *In vitro* studies indicated a decline in the population of reactive astrocytes and a suppression in the transcriptional activity of pro-inflammatory cytokines and MCP-1 upon Gap19 treatment. Interestingly, while Gap19 decreases Cx43 protein expression in astrocytes, it does not appear to impact its transcriptional activity. The degradation of Cx43 might be orchestrated via the ubiquitin-proteasome pathway (Yu et al., 2020).

Yes-associated protein (YAP) functions as a regulator of cell-to-cell communication and binds to Cx43. YAP, expressed in astrocytes, exhibits activity modulation based on its phosphorylation status. After hemin stimulation, there is an observed upregulation in phosphorylated YAP. Furthermore, Gap19 triggers both nuclear YAP expression and its translocation, leading to reduced cyclic YAP levels. These findings hint at the potential of Gap19 in curbing the pro-inflammatory attributes of reactive astrocytes through YAP activity modulation (Yu et al., 2020). The regulation of suppressor of cytokine signaling 1(SOCS1) and SOCS3 falls under the purview of the YAP signaling cascade (Huang et al., 2016). Elevated expression of SOCS1 and SOCS3 curbs the TLR4-NFκB and Janus kinase- signal transducer and activator of transcription (JAK-STAT) pathways (Cianciulli et al., 2017). In *in vitro* scenarios, Gap19 augments SOCS1 and SOCS3 expression while downregulating markers associated with TLR4, p-IKKβ, p-p65, p-JAK2, and p-STAT3. This implies that Gap19 modulates the Cx43-YAP-SOCS1/SOCS3 axis in reactive astrocytes, inhibiting pathways like TLR4-NFκB and JAK2-STAT3. A blockade in nuclear YAP activity nullifies the neuroprotective impacts of Gap19 on brain injury (Yu et al., 2020). It can be seen that the bidirectional communication between astrocytes and neurons plays an important role in improving secondary injury after cerebral hemorrhage.

#### 3.2.5 Lactate

Astrocyte-neuron lactate shuttle (ANLS) suggests that glutamate released from neuronal activity into synaptic gaps can be absorbed by astrocytes and trigger glucose uptake, which is then converted into lactate and used by neurons through monocarboxylate transporters (Tarczyluk et al., 2013). In clinical practice, High lactate to albumin ratio (LAR) is associated with increased hospitalization and ICU mortality rates in patients with cerebral hemorrhage (Wu et al., 2023). Zhou et al. (2018)'s experiment showed that after cerebral hemorrhage, lactate accumulates around the hematoma, participates in the expression of growth factors involved in angiogenesis and nerve regeneration, and reduces cell death. The mechanism may be related to the activation of NF-κB signaling pathway is associated with enhancing angiogenesis and neurogenesis (Zhou et al., 2018).

This suggests that the neuroprotective effect of lactate may be a potential therapeutic target for promoting brain repair after ICH. Further research on astrocytes and lactate metabolism can contribute to the treatment.

## 4 Conclusion

**Figure 1:**
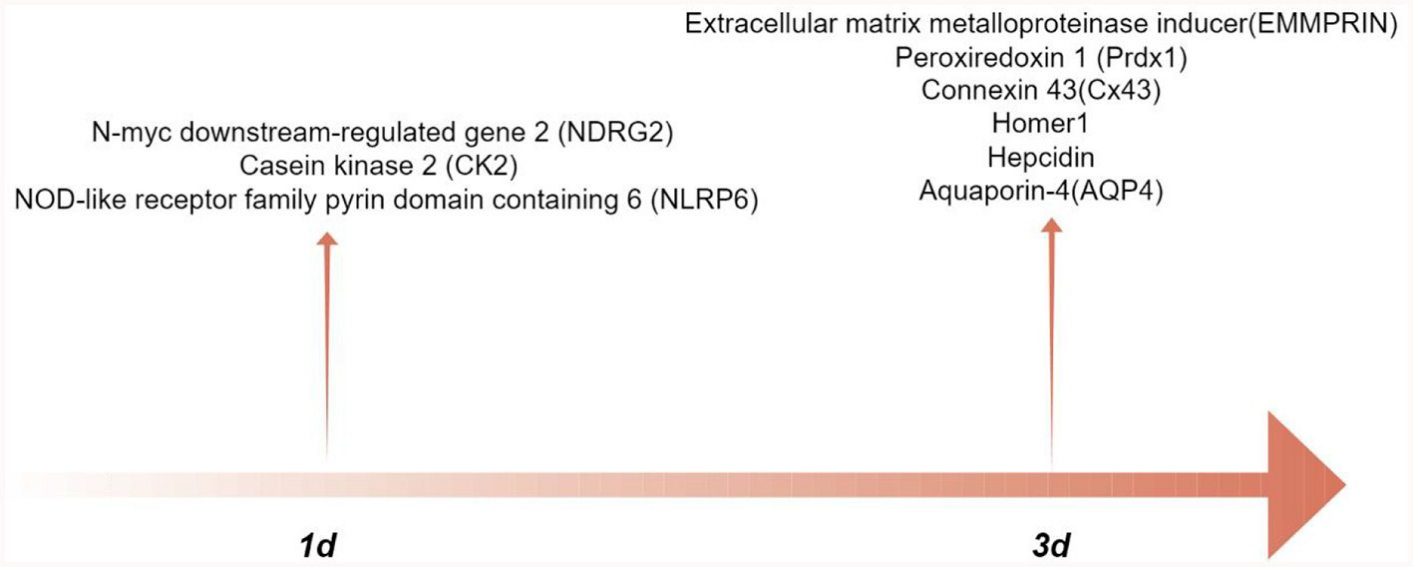
The peak-time of astrocyte proteins expression after intracerebral hemorrhage (by Figdraw.).

Astrocytes are engaged in a number of pathogenic alterations following ICH-induced secondary injury. In intracerebral hemorrhage, changes in the expression of astrocyte related proteins play a role in inflammatory response, brain edema, cell apoptosis, oxidative stress, and neuronal energy metabolism (Table 1). In addition, Figure 1 illustrates the peak expression of proteins located in astrocytes. After intracerebral hemorrhage, microglia are activated, and astrocytes can interact with microglia. By observing cellular markers, individual proteins can affect the phenotype of microglia and regulate their function. However, there is still a lack of relevant evidence on how astrocytes control the activation and function of microglia after cerebral hemorrhage. The research on astrocyte reactivity and cellular phenotype transformation after cerebral hemorrhage is still incomplete. Phenotypic transition may help alleviate brain damage; however, the precise signaling mechanism and timing of phenotypic transition remain unknown. In addition, some scholars believe that the naming convention for astrocytes A1/A2 should describe the response state of astrocytes based on molecular expression patterns and functional changes (Lawrence et al., 2023).

**Table 1:** The effect of astrocyte protein in intracerebral hemorrhage.

**Protein**	**Mechanism involved**	**Signal pathway involved**
S100B (↓)	1. IL-1β, TNF-α (↓) 2. ROS (↓)	—
NLRP6 (↑)	IL-1β, IL-6, TNF-α (↓)	TLR4 pathway
Prdx1 (↓)	1. IL-6, TNF-α (↓) 2. Apoptosis (↓) 3. Target mRNA stability	—
TLR2 (↓)	1. MMP (↓) 2. BBB damage (↓) 3. Neutrophil infiltration (↓)	Heme-TLR2 pathway
CK2 (↑)	1. IL-6, TNF-α (↓) 2. Neuronal apoptosis (↓) 3. Oxidative stress (↓) 4. Regulate NR2B phosphorylation and NR2B-PSD95 complex	—
AQP2 (↓)	IL-1β (↓)	TLR4/NFκB-P65 pathway
AQP4 (↑)	1. BBB damage, ROS (↓) 2. Protect astrocyte and tight junction	JNK and ERK pathway
AQP4 (↓)	Cerebral edema (↓)	NF-κB pathway
AQP9 (↓)	Cerebral edema (↓)	NF-κB pathway
Hepcidin (↓)	The accumulative iron in the brain, the brain water contents (↓)	TLR4/MyD88 pathway
NDRG2 (↓)	1. Hematoma volume (↓) 2. Neuronal apoptosis (↓)	NF-κB/GLT1 pathway
GHK (↑)	1. miR-339-5p (↓) 2. Apoptosis (↓)	miR-339-5p/VEGFA pathway
EMMPRIN (↓)	1. MMP-9 (↓) 2. Neutrophil infiltration (↓)	—
TRAP1 (↓)	1. IL-1β, IL-6 (↓) 2. p-ERK, p-ERK/ERK, p-IKKβ/IKKβ (↓) 3. NOX1, Calpain1 (↓)	MAPK/NF-κB pathway
HN (↑)	1. PPARγ/Mn-SOD (↑) 2. IL-1β (↓)	—
Cx43 (↓)	1. Apoptosis (↓) 2. Inflammation (↓)	—

Some articles only explain the activation of astrocytes through the expression of glial fibrillar acidic protein (GFAP), or explain the impact of proteins on secondary injury after cerebral hemorrhage through changes in astrocyte related proteins after cerebral hemorrhage. However, there is a lack of clear mechanisms to explain how these proteins directly regulate the state of astrocytes. In future research, exploration can be conducted based on different cell subpopulations or molecular expression patterns to improve the function of astrocytes in different stages of cerebral hemorrhage. In the bidirectional communication between astrocytes and neurons after cerebral hemorrhage, research mainly focuses on glutamate and mitochondria. Research based on the neuroprotective effect of lactate may be a potential therapeutic target for treating neurological dysfunction after cerebral hemorrhage. Part of the pathways affect the reactivity of astrocytes and the inflammatory response after cerebral hemorrhage. Future research can explore the mechanisms by which these pathways affect astrocytes during cerebral hemorrhage. A comprehensive exploration of astrocytes has the potential to improve therapeutic outcomes. Some astrocyte proteins continue to play a contentious function, which may be attributable to varying protein expression levels at various timepoints following the onset of the disease. To identify more effective treatment targets, it is imperative to conduct a detailed investigation into the interactions among diverse proteins. Examining these aspects is crucial for understanding ICH astrocytes.

## Data availability statement

The datasets presented in this study can be found in online repositories. The names of the repository and accession number can be found below: https://www.ncbi.nlm.nih.gov/bioproject/PRJNA1039505.

## Author contributions

HD: Conceptualization, Writing—original draft. XW: Writing—review & editing. B-WZ: Writing—review & editing. ZW: Writing—review & editing. WZ: Conceptualization, Writing—review & editing.
